# Cost of physician-led home visit care (Zaitaku care) compared with hospital care at the end of life in Japan

**DOI:** 10.1186/s12913-016-1961-x

**Published:** 2017-01-17

**Authors:** Kentaro Kinjo, Tomoko Sairenji, Hidenobu Koga, Yasuhiro Osugi, Shin Yoshida, Hidefumi Ichinose, Yasunori Nagai, Hiroshi Imura, Jeannette E. South-Paul, Mark Meyer, Yoshihisa Honda

**Affiliations:** 1 Department of General Medicine, Morinosato Hospital/Kameda Hospital, Tokai University, Keio University, 3-1-1 Morinosato, Atsugi, Kanagawa 243-0122 Japan; 2 Department of Family Medicine, University of Washington, E304 Health Sciences 1959 NE Pacific Street, Seattle, WA 98195-6390 USA; 3 Department of Medical Information Analysis, Iizuka Hospital, 3-83 Yoshiomachi, Iizuka, Fukuoka 820-8505 Japan; 4 Department of General Medicine, Fujita Health University, 1-98 Kutzukake Dengakugakubo, Toyoake, 470-1192 Japan; 5 Department of General Medicine, Aso Iizuka Hospital, Iizuka Kaita Family Medicine Residency Program, 3-83 Yoshiomachi, Iizuka, Fukuoka 820-8505 Japan; 6 You-no-mori clinic, 444-1 Bepucho, Matsuyama, Ehime 791-8056 Japan; 7 Department of Family Medicine, University of Pittsburgh, 4420 Bayard Street, Suite 520, Pittsburgh, PA 15260 USA; 8 Kaita Hospital, 1061 Haranokuchi, Iizuka, Fukuoka 820-1114 Japan

**Keywords:** Medical costs, Home visit, End of life care, Zaitaku care, Physician-led team home visit, Japan

## Abstract

**Background:**

Physician-led home visit care with medical teams (Zaitaku care) has been developed on a national scale to support those who wish to stay at home at the end of life, and promote a system of community-based integrated care in Japan. Medical care at the end of life can be expensive, and is an urgent socioeconomic issue for aging societies. However medical costs of physician-led home visits care have not been well studied. We compared the medical costs of Zaitaku care and hospital care at the end of life in a rapidly aging community in a rural area in Japan.

**Methods:**

A cross-sectional study was performed to compare the total medical costs during patients’ final days of life (30 days or less) between Zaitaku care and hospital care from September 2012 to August 2013 in Fukuoka Prefecture, Japan.

**Results:**

Thirty four patients died at home under Zaitaku care, and 72 patients died in the hospital during this period. The average daily cost of care during the last 30 days did not differ significantly between the two groups. Although Zaitaku care costs were higher than hospital care costs in the short-term (≦10 days, Zaitaku care $371.2 vs. Hospital care $202.0, *p* = 0.492), medical costs for Zaitaku care in the long-term care (≧30 days) were less than that of hospital care ($155.8 vs. $187.4, *p* = 0.055).

**Conclusions:**

Medical costs of Zaitaku care were less compared with hospital care if incorporated early for long term care, but it was high if incorporated late for short term care. For long term care, medical costs for Zaitaku care was 16.7% less than for hospitalization at the end of life. This physician-led home visit care model should be an available option for patients who wish to die at home, and may be beneficial financially over time.

**Electronic supplementary material:**

The online version of this article (doi:10.1186/s12913-016-1961-x) contains supplementary material, which is available to authorized users.

## Background

By 2050, the population of elderly people (those over 65 years of age) is expected to grow to 38.8% in Japan, 37.4% in Korea, 36.4% in Spain, 24.1% in United Kingdom (U.K.) and 21% in the United States (U.S.) [1]. Creating a sustainable medical system for the growing elderly population is a looming challenge for many of these countries. Japan’s elderly already comprises of 25.0% (2014) of the nation’s population [2], with higher percentages in some communities that are even further along the aging curve. These communities have already implemented measures that may prove to be a model of care for others. Kaita, a rural area in Fukuoka prefecture is one of these locations, with 34.2% of its 5,914 inhabitants over 65 years of age (2014) [3].

In Japan, all citizens have medical care and long-term care coverage under a universal health insurance system, that consists of occupational insurance for salaried workers (employees) and National Health Insurance (NHI) for self-employed, including farmers and the elderly [4]. To address the aging population with one of the lowest total fertility rates in the world (1.39 in 2010) [4], the Japanese government introduced the Long-Term Care Insurance (LTCI) system to supplement the existing universal health insurance and pension system in 2000 [4–7]. This system was implemented to promote socialization of care with the slogan “Transitioning From Care by Family to Care by Society” [4, 5]. Another goal of the LTCI is to support organized community-based integrated care [8, 9], especially for the increasing number of the frail elderly and home-bound patients with medical and nursing needs [5–7].

LTCI supports care services, community-based services, and in-facility services [4]. Although all primary insured persons (over age 65) are candidates for care, secondary insured persons (ages 40-65) diagnosed with 15 diseases (Alzheimer’s disease, stroke, end-stage cancer, etc.) can also utilize care services. Those under 40 are not eligible and are not required to pay the LTCI fee. When insured persons need to use the LTCI, they submit a request to the municipal government for their primary physicians to assess and evaluate their physical and mental status. A local Nursing Care Needs Certification Board determines the eligibility and care needs level for insured people using these results. Evaluated persons are categorized as “rejected”, “care support level” 1 ~ 2, and “care level” 1 ~ 5, which corresponds to benefit eligibility ranging from $417 to $3005 per month in 2012 and 2013. Care services using allotted benefits are coordinated by a care manager in discussion with the insured person and his/her family [4–7].

The LTCI budget is derived from 50% tax (25% state, 12.5% prefecture, and 12.5% municipality) and 50% insurance contribution from subscribers, and is controlled by the municipal government (insurer). The municipal government pays 90% and subscribers pay 10% for billed services. Insurance contributions to the municipal government are deducted from the pension of the primary insured person (ages 65 and older) and from the public medical insurance premium for the secondary insured person (ages 40 to 64) subscribers [4].

Although home care services and programs have also been implemented in other countries [10–16], Zaitaku (“staying at home” in Japanese) care is unique in that primary care physicians lead home care teams consisting of nurses, rehabilitation staff, pharmacists, medical aides (helpers), care managers, and case managers using LTCI and public medical insurance including national health insurance (NHI) [8]. To be enrolled in Zaitaku care, patients must be unable to get to outpatient clinic and must reside within roughly 16 km of the hospital or clinic that provides these services. They must pay a monthly management fee to contract for medical care. As part of this contract, physicians are required to see patients once or twice per month depending on their medical needs. Following enrollment in the program, a visiting nurse triages the patient to determine whether a physician (either the primary care physician or the on-call physician at night or on weekends) is needed. Primary care physicians care for a broad range of patients at home, from infants to elderly patients with complex medical problems. Teams can provide services such as phlebotomy, ultrasounds, fluid aspiration (from abdomen, chest and knees), intravenous infusions, antibiotics, respiratory therapy, home oxygen therapy, physical therapy, occupational therapy, pharmacotherapy education, and palliative care.

Dying at home was the tradition for many years in Japan. Eighty three percent of the population died at home in 1951. Current literature on end of life care suggests that people still prefer to stay at home [17–21], but more Japanese citizens die in health care facilities (81.6% in 2003) than at home [8]. There are still some roadblocks such as deficiencies in patient and family knowledge regarding home care and support systems for caregivers, but the biggest barrier may be the lack of widespread medical systems that can adequately support patients’ wishes to spend their last days at home [22, 23].

With the aging population, it is essential that the home care system is financially sustainable. Some reports show that home care can generally save 19%-25.5% of medical costs, but there are scant data to show that there is a cost-benefit for home visits compared to standard care [24–30]. For example, team-managed home-based primary care costs are 6.8% higher at 6 months and 12.1% higher at 12 months more than control [31]. End of life care, with completion of advance directives and incorporating palliative care at home, showed a 45% savings by reducing emergency admission and hospitalizations [32–34], but other studies failed to show that home palliative care service had positive cost benefits overall [35, 36]. Most existing studies focus only on nursing care costs, because medical costs are decided by the conventional reimbursement system with public medical insurance systems and/or medical insurance companies. Therefore, in this report, we examined total medical costs including medical and nursing care fee for home visit care hoping to create a milestone for other aging societies. We did not assess the family’s additional cost- food, shelter, clothing, etc.

Although some studies assess medical costs at the end of life comparing home-based and hospital-based palliative care [32–36], no study has specifically examined the total medical costs of physician-led team home visit care at the end of life compared to hospital care. This report describes a cross-sectional study of medical costs for physician-led team home visit care vs. in-hospital management at the end of life.

## Methods

### Overview of study design

Kaita Hospital has an outpatient clinic and 96 inpatient beds, located in Iizuka city, Fukuoka prefecture, Japan. Seven supervising family medicine physicians (attendings) and 3 family medicine residents provided inpatient and outpatient services to patients and supervised a physician-led team home visit (Zaitaku) care team in 2012 and 2013. After patients were enrolled in the Zaitaku care with or without LTCI, care managers coordinated their care services. The Zaitaku care team was of standard composition: family medicine physicians, nurses, care managers, and medical aides (home helpers). Physical therapists, occupational therapists and pharmacists were incorporated as needed. Team communication occurred though meetings, internet communication tools, phone calls and fax.

Medical charts and total medical costs for medical and nursing care of all patients who died at home with Zaitaku care or in the hospital during the last 30 days of life were analyzed as a cross-sectional study from September 2012 to August 2013 at Kaita hospital and home care facilities.

#### Participants

Patient profiles were collected for Zaitaku and hospital care during the period. The two groups were identified by gender, whether they had cancer or dementia, usage of opioids or IV fluids, oxygen requirements, and antibiotics treatment status (Table 1). Patients under Zaitaku and hospital care were further divided into 3 groups depending on the duration of home care before death (short-term defined as 1 to 10 days, medium-term defined as 11 to 29 days, and long term defined as more than 30 days, Table 5) and sorted by factors using the same methods as in Table 1.

**Table 1:** Baseline characteristics of patients utilizing Zaitaku and hospital care

Characteristics	Zaitaku care (*n* = 33)	Hospital care (*n* = 72)	*p*-value
Age (mean ± SD)	80.7 ± 12.5	80.2 ± 9.5	0.571**
Gender			1.000*
Female (%, n)	39.4% (*n* = 13)	40.3% (*n* = 29)	
Male (%, n)	60.6% (*n* = 20)	59.7% (*n* = 43)	
Primary diagnosis (%, n)			
Cancer	66.7% (*n* = 22)	45.8% (*n* = 33)	0.060*
Dementia	39.4% (*n* = 13)	40.3% (*n* = 29)	1.000*
Oxygen (%, n)	36.4% (*n* = 12)	72.2% (*n* = 52)	0.001*
Infusion fluid (%, n)	84.8% (*n* = 28)	94.4% (*n* = 68)	0.136*
Opioids (%, n)	42.4% (*n* = 14)	40.3% (*n* = 29)	0.834*
Antibiotics (%, n)	27.3% (*n* = 9)	40.3% (*n* = 29)	0.274*

*p-values were calculated using analysis of the Fisher’s exact test

**p-value was calculated using analysis of the Wilcoxon rank sum test

#### Medical costs

Total medical costs (including public medical insurance and LTCI) were claimed by the hospital or/and care facilities. This included medical fees under public insurance, LTCI fees for services, nursing services, pharmacist medication-management, medical aids, physical and occupational therapy with or without transportation fees. Medical costs for physician services, tests, and medications were calculated as itemized treatments, which were coded by the name of disease in a system similar to ICD10, for reimbursement under the public medical insurance (Additional files 1 and 2). Episodic medical and nursing fees for conditions such as severe decubitus ulcers, need for IV fluids, and palliation of end-stage cancer were covered by public medical insurance and other care by LTCI system. Terminal care fees were claimed when physicians diagnosed patients’ death at home and visited more than twice within 14 days and/or within 24 h of their death. Medical contract fees were specific for Zaitaku care, and mandated periodic physician home visits periodically (usually twice a month), and covered communication expenses, medical equipment and simple procedures such as collecting sputum samples and simple dermatologic care [37]. For the purposes of comparison, medical costs were calculated as daily rates instead of the total cost for 30 days prior to death. Currency conversion from Japanese yen to U.S. dollars was performed by using the U.S. dollar equivalent rate in 2015 (1U.S. dollar = 120 Japanese yen).

Total medical costs of patients utilizing Zaitaku and hospital care were analyzed (Table 2) and compared those two groups by factors (age, sex, cancer, dementia, opioid use, infusion fluid, oxygen (Tables 1, 3, and 5). Patients receiving hospital care were further divided into two groups, depending on whether they had previously received home visit care or not. Medical costs of hospital care were compared after or without Zaitaku care (Table 4). Total medical costs were compared with national average medical costs in Japan (Fig. 2, Additional file 3) [38]. The medical costs of patients under Zaitaku and hospital care of short (≦10 days), medium (11-29days), long (≧30 days)-term periods were compared (Table 6).

**Table 2:** Total medical costs of patients utilizing Zaitaku and hospital care (US$/day)

Characteristics	Zaitaku care	Hospital care	*p*-value
Total medical costs	371.2 (*n* = 33)	202.0 (*n* = 72)	*p* = 0.492*

*p-value was calculated using analysis of the Wilcoxon rank sum test

**Table 3:** Medical costs of Zaitaku and hospital care by using propensity score method (US$/day)

Characteristics	Zaitaku care (*n* = 33)	Hospital care (*n* = 33)	*p*-value
Medical costs median [Q1, Q3]	201.2 [151.8, 749.0]	188.2 [164.6, 229.3]	0.60*

*p-value was calculated using analysis of the binomial logistic regression

**Table 4:** Medical costs of hospital care after or without Zaitaku care (US$/day)

Characteristics	Hospital care		*p*-value
	After Zaitaku care (*n* = 17)	Without Zaitaku care (*n* = 55)	
Medical costs	200.8	202.2	0.952*

*p-value was calculated using analysis of the Wilcoxon rank sum test

#### Relationship between medical costs and home visiting times

**Fig. 1:**
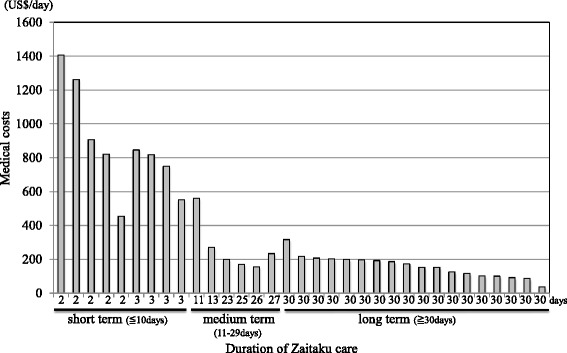
Total cost of Zaitaku care at end of life. Horizontal axis shows the duration of Zaitaku care. Vertical axis shows the medical costs per day for each patient. The patients are plotted by the shorter duration and the higher costs of Zaitaku care. Those data are shown in Additional file 1 and 2

Medical costs and home visiting times were analyzed for the different care term groups (Table 7, Fig. 1).

### Statistical analyses

Values were expressed as the mean ± standard deviation when the data were normally distributed data or the median ± interquartile range when the data did not follow a normal distribution. Fisher’s exact test was used for the categorical baseline variables of patient profiles, and the non-parametric Wilcoxon rank sum test and Kruskal-Wallis test were used for age analysis (Tables 1 and 5). Medical costs were compared using the Wilcoxon rank sum test (Tables 2, 3, 4, and 6). Propensity scores were generated to estimate the probability of home visit care or hospital care assignment by using binomial logistic regression. The variables for estimating propensity scores included age, sex, cancer, opioids, infusion fluid, oxygen and dementia. A two-tailed p value <0.05 was considered significant by the non-parametric Wilcoxon rank sum test (Table 3). The comparison between Zaitaku care with monthly fees or adjusted monthly fees and hospital care were analyzed using multiple comparison (Bonferroni) methods (Table 6). Medical costs and home visits numbers for Zaitaku were compared between those groups using non-parametric Kruskal-Wallis test (Table 7).

**Table 5:** Baseline characteristics of patients utilizing Zaitaku and hospital care for short (≦10 days), medium (11-29days), long (≧30 days)-term periods

	Zaitaku care				Hospital care			
Characteristics	Short-term (*n* = 9)	Medium-term (*n* = 7)	Long-term (*n* = 17)	*p*-value	Short-term (*n* = 13)	Medium-term (*n* = 16)	Long-term (*n* = 43)	*p*-value
Age (mean)	71.0 ± 15.5	82.3 ± 6.1	85.5 ± 10.3	0.024**	74.9 ± 10.7	83.8 ± 9.0	80.5 ± 8.8	0.008**
Gender				0.011*				0.529*
Female (%, n)	66.7% (*n* = 6)	14.3% (*n* = 1)	35.3% (*n* = 6)		46.2% (*n* = 6)	50.0% (*n* = 8)	34.9% (*n* = 15)	
Male (%, n)	33.3% (*n* = 3)	85.7% (*n* = 6)	64.7% (*n* = 11)		53.8% (*n* = 7)	50.0% (*n* = 8)	65.1% (*n* = 28)	
Diagnosis (%, n)								
Cancer	66.7% (*n* = 6)	71.4% (*n* = 5)	64.7% (*n* = 11)	1.000*	76.9% (*n* = 10)	37.5% (*n* = 6)	39.5% (*n* = 17)	0.057*
Dementia	33.3% (*n* = 3)	28.6% (*n* = 2)	47.1% (*n* = 8)	0.715*	15.4% (n = 2)	56.2%(n = 9)	41.9% (*n* = 18)	0.075*
Oxygen (%, n)	33.3% (*n* = 3)	42.9% (*n* = 3)	35.3% (*n* = 6)	1.000*	61.5% (*n* = 8)	87.5% (*n* = 14)	69.8% (*n* = 30)	0.271*
Infusion fluid (%, n)	88.9% (*n* = 8)	71.4% (*n* = 5)	88.2% (*n* = 15)	0.675*	84.6% (*n* = 11)	87.5% (*n* = 14)	100.0% (*n* = 43)	0.023*
Opioids (%, n)	66.7% (*n* = 6)	28.6% (*n* = 2)	35.3% (*n* = 6)	0.303*	76.9% (*n* = 10)	37.5% (*n* = 6)	30.2% (*n* = 13)	0.011*
Antibiotics (%, n)	22.2% (*n* = 2)	42.9% (*n* = 3)	23.5% (*n* = 4)	0.595*	7.7% (*n* = 1)	43.8% (*n* = 7)	48.8% (*n* = 21)	0.022*

*p-values were calculated using analysis of the Fisher’s exact test

**p-value was calculated using analysis of the non-parametric Kruskal-Wallis test

**Table 6:**
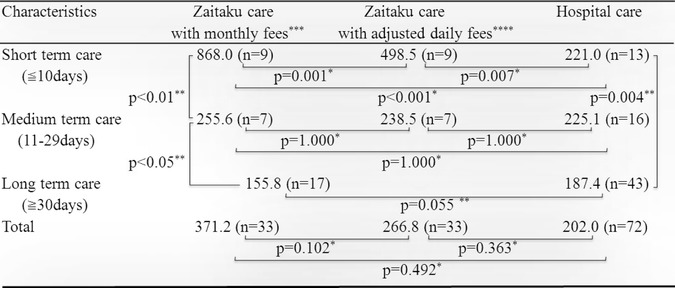
Total medical costs of patients utilizing Zaitaku with monthly fees, adjusted daily fees and hospital care for 30 days

^*^ *p*-values were calculated using multiple comparison (Bonferroni) method

^**^ *p*-value was calculated using analysis of the Wilcoxon rank sum test

^***^Monthly fees include the monthly contract fee and the one-time terminal care fee for the final day of life

^****^Adjusted fees include the adjusted daily contract fee and the one-time terminal daily care fee for the final day of life

**Table 7:** Number of visits per day for Zaitaku Care (times/day)

	Zaitaku care			
Characteristics	Short-term care (≦10 days)	Medium-term care (11-29 days)	Long-term care (≧30 days)	*p*-value
Number of visits /day	1.48 (*n* = 9)	0.50 (*n* = 7)	0.27 (*n* = 17)	*p* < 0.0001*

*p-value was calculated using analysis of the the non-parametric Kruskal-Wallis test

All statistical analyses were performed by SAS version 9.4.

## Results

### Patient characteristics

From September 2012 to August 2013, 34 patients died at home under Zaitaku care, and 72 patients died under hospital care. All patients completed their advance directives at the time of admission to either program. For Zaitaku care patients, total medical costs were calculated including public medical insurance, LTCI and medication costs, except for one patient whose LTCI costs could not be obtained. Among 33 patients who died under Zaitaku care, three patients switched from hospital care to Zaitaku care (No. 8, 11, 29 of Additional files 1 and 2). One patient was hospitalized between days 4 to 8 before death (No.33, Additional file 2). Among 72 patients who died under hospitalized care, 17 patients had switched from Zaitaku care. For patients who died in the hospital, total cost for hospital care was obtained. The mean age was 80.7 years (range, 36-98 years) for Zaitaku care and 80.2 years (range, 51-98 years) for hospital care (*p* = 0.571, Table 1). Males comprised 60.6% of the Zaitaku care group and 59.7% of the hospital care group (*p* = 1.000). Cancer patients represented 66.7% of the Zaitaku care group and 45.8% of the hospital care group (*p* = 0.060). There was no difference in the proportion of dementia patients (*p* = 1.000), those on opioids (*p* = 0.834), those receiving IV fluids (*p* = 0.136), or those with antibiotic use (*p* = 0.274) in each group. Oxygen was more frequently used in hospitalized patients compared with those receiving Zaitaku care (*p* < 0.01) (Table 1).

### Comparison of medical costs within 30 days before dying

The medical cost of Zaitaku care and hospital care within 30 days prior to death were calculated. The average medical cost for Zaitaku care (33 cases) was $371.2/day. The average medical cost for hospital care (72 cases) was $202.0/day. Zaitaku care cost more than hospital care, but the difference was not significant (*p* = 0.492, Table 2). To match patients in Zaitaku care and hospitalized care, the propensity score method was used to compare medical costs between matched patients with Zaitaku care and hospitalized care. There was no significant difference between those groups (*p* = 0.60, Table 3).

### Costs for hospital care after or without Zaitaku care within 30 days of dying

The average total medical cost of hospital care for the 30 days preceding death was $202.0/day (72 cases, Table 2). The average cost of hospital care without any Zaitaku care (55 cases) was $202.2/day, and the cost of hospital care after some Zaitaku care (17 cases) was $200.8/day (Table 4, *p* = 0.952). Thus, there was no significant difference in the medical costs with or without some Zaitaku care preceding the hospital care at final day of life.

### Patient characteristics for short, medium, and long-term periods

Patient characteristics of short (≦10 days), medium (11-29days), long (≧30 days)-term periods were examined by age, gender, diagnosis (cancer and/or dementia), oxygen, infusion fluid, opioids, and antibiotic use (Table 5). In both Zaitaku and hospital care, age of the short-term group was significantly younger than other groups (*p* = 0.024 at Zaitaku care, *p* = 0.008 at hospitalized care) and used opioids more than other groups (*p* = 0.303 at Zaitaku care, *p* = 0.011 at hospitalized care). With the hospital care group, short-term users had more cancer (*p* = 0.057) and less dementia (*p* = 0.075), used less antibiotics (*p* = 0.022), infusion fluid (*p* = 0.023). With the Zaitaku care group, there was no difference diagnoses among those periods (cancer:*p* = 1.000, dementia:*p* = 0.715), or use of antibiotics (*p* = 0.595) and infusion fluids (*p* = 0.303).

### Costs for different durations of Zaitaku and hospital care within 30 days of dying

The average medical cost of Zaitaku care for short-term was $868.0/day (9 cases), medium-term was $255.6/day (7 cases), and long-term was $155.8/day (17 cases) (Table 6). Short-term Zaitaku care costs were significantly higher than medium-term care (Table 6, p < 0.01) and medium-term care were significantly higher than long-term care (Table 6, *p* < 0.05). Average medical costs of Zaitaku care for more than 90 days was less ($131.0/day) than the care of 30-90 days ($150.5/day, Additional files 1, and 2). The average medical cost of hospital care for short-term was $221.0/day (13 cases), medium-term was $225.1/day (16 cases), and long-term was $187.4/day (43 cases) (Table 6). Among those groups, medical costs for shorter care was significantly higher (Table 6, *p* = 0.004). The average medical costs of Zaitaku and hospital care for short, medium, and long-term period were compared (Table 6). Zaitaku care fees included the monthly contract fee and the one-time terminal care fee for the final day of life. With short-term care, Zaitaku care was higher than that of hospital care (868.0 vs. 221.0 *p* < 0.001). With long-term care, Zaitaku care was less than that of hospital care (155.8 vs. 187.4 *p* = 0.055).

To investigate the underlying cause for the higher costs of short-term Zaitaku care the daily medical costs with adjusted daily additional fees (divided care days from the monthly contract fee and the one-time terminal care fee for the final day of life) were calculated (Table 6): $498.5/day (42.6% less with adjusted daily additional fees) for short-term, and $238.5/day (6.7% less with adjusted daily additional fees) for medium-term (Additional files 1, and 2). Total average medical costs of Zaitaku care within 30 days adjusting those fees is $266.8 (28.2% less with adjusted daily additional fees). It was 1.32 times higher than the cost of hospitalization at the end of life, but not a significant difference (*p* = 0.3625, $266.8 vs. $202.0, Table 6).

The relationship between the number of physicians’ visits per day and medical costs were also examined (Table 7). The numbers of visits provided for short-term care was significantly higher than medium-term care and longer care (*p* < 0.0001).

## Discussion

Medical costs at the end of life pose serious concerns for aging societies. In the U.S., 25.1% of medical insurance costs are accrued in the last year of life [39]. In the U.K., approximately 20% of hospital bed days are utilized for end-of-life care [40]. We showed that Zaitaku care cost more than hospital care for short-term care (≦10 days) (*p* < 0.01, Table 6), but Zaitaku care for long-term care (≧30 days) might be associated with a 16.7% cost savings compared with exclusively hospital care (*p* = 0.0549, Table 6). The average duration of Zaitaku is 108.5 days. Longer Zaitaku care more than 90 days generated less costs ($131.0/day) than the care of 30-90 days ($150.5/day, Additional files 1, and 2).

**Fig. 2:**
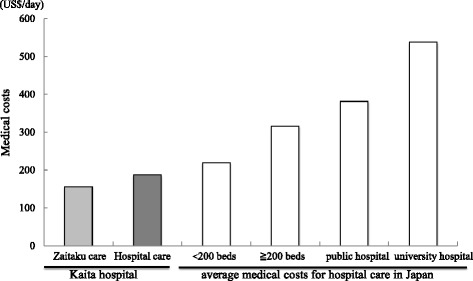
Medical costs of Zaitaku and hospital care vs. average hospital care in Japan. Comparing the medical costs of Zaitaku and hospital care at the end of life (30 days) at Kaita hospital and the average medical costs in Japanese hospitals. The costs of Zaitaku care (left light gray column, Kaita hospital, long term, $155.8) and hospital care (left dark gray column, Kaita hospital:$187.4; white columns, Japanese average <200 beds: $219.8, Japanese average ≧200 beds: $316.1, Japanese average of public hospital: $381.4, Japanese average of university hospital: $537.7, ref. 38, Additional file 3) were shown

The Japanese national average costs for hospital care is $219.8/day at small hospitals with less than 200 beds, $316.1/day at hospitals with more than 200 beds, $381.4/day at public hospitals, and $537.7/day at university hospitals in 2012 and 2013 (Fig. 2, Additional file 3) [41]. The average medical cost in our hospital care even at the end of life care was $202.0/day, that was lower than at any public or university hospital in Japan. These lower costs relate to our lower ratio of nurses to patients (1 to 10) and the fact that most of the patients had advanced care planning. The medical costs in our hospital were lower than the average in Japan, and the cost of Zaitaku care for more than 30 days at the end of life may also be found to be less costly (Table 6).

We found that short-term Zaitaku physician-led home visit care for end of life was costly (Table 6, Fig. 1, Additional files 1, and 2). Some contributing factors may be the monthly contract fee for the Zaitaku team, one-time terminal care fee for the final day of life care at home specifically for Zaitaku care from the Japanese Ministry of Health, Labor and Welfare (JMHLW), monthly payments for care services, and costs of home care equipment including home oxygen. Our Zaitaku care facility is recognized as a superior hospital by the JMHLW, with more than 3 physicians who provide more than 10 emergency home visits and oversee more than 4 patient deaths at home per year. To assess the medical costs for Zaitaku (physician-led home visits team) care, total medical costs, the daily terminal care fee, and daily medical care contract fee, which are specific to the Zaitaku care by the JMHLW, were all calculated. The total average medical cost of Zaitaku care with adjusted daily fees was 28.2% less than monthly fees (Table 6) and the cost without monthly fees was 14% less than hospital care within 30 days of the last day (Additional files 1 and 2).

Frequent visits for Zaitaku care in the short term may result in higher costs than in the longer term (Table 7), because every physician-led home visit was paid from public insurance. The findings of high costs of Zaitaku care in the short period are valid. It is likely that subsequent work will identify differences between patients cared for primarily in hospital and those at home – not just relative to diagnosis (since the diagnoses between the two groups were similar) – but likely related to other as yet unidentified factors. As more patients survive severe illnesses that previously would have resulted in rapid deaths, we will need to identify how to appropriately care for them in the post-hospitalization period in a compassionate and cost-effective fashion.

Our certified family physician group examined medical costs of a physician-led home visit team care during the last 30 days of life. In our practice, the same group of primary care physicians provides medical care with palliative measures at the end of life both at home and at the hospital. The fact that we are able to compare these two groups of patients cared for by one consistent group of physician providers is a strength of this study. Physicians learned palliative care from lectures and workshops for cancer patients and end of life care at a 2-day program of the Palliative Care Emphasis program on symptom management and Assessment for Continuous medical Education (PEACE) project [41] guided by experts from the U.S. and the U.K. Physicians and nurses offer the palliative care supporting patients and their families with Zaitaku team members.

Some patients did not use LTCI (Additional file 2, case No. 22, 28, 33), which lowered the total cost of their medical care. The costs for food and nursing care by family members or volunteers for patients receiving Zaitaku care were not included, which may decrease the overall costs compared to the hospital care group. For example, the medical cost for one patient with amyotrophic lateral sclerosis (Additional file 2, No. 33) was the lowest in the Zaitaku care group. That patient’s family supported the patient for a long time, and they did not want to utilize care services.

One significant benefit of Zaitaku care is that it is an interdisciplinary, team-based approach. Various members of the team help each other to prevent provider burnout [29, 31]. The Zaitaku team can provide essential physical and emotional support to caregivers by valuing their efforts and helping alleviate caregiver fatigue. This can additionally elevate both the patients’ and families’ satisfaction at the end of life [21, 31]. The comprehensive team approach also makes problem-solving easier compared to teams with only physicians or nurses. Those benefits help fulfill patients’ and their caregivers’ wishes to spend their last days at home.

Although there was no significant difference between the groups in regards to sex or age, there were more cancer patients in the Zaitaku care group compared with the hospital care (*p* = 0.06, Table 1). Patients with cancer have significant health care needs and may need comprehensive assessment of prognosis to appropriately determine the level of care as they plan their end of life care at their home. Their families might support their wishes given their limited remaining time [42].

Our study has limitations. (1) The study is a pilot and thus is small; (2) all costs are not accounted for because we didn’t assess the non-medical costs to the families; (3) the time analysis was limited and focused on the last 30 days of care; (4) many social and insurance issues that support Zaitaku care are unique to Japan, but could be adapted in other countries; (5) the reimbursement fee schedule for medical costs is adjusted every two years by JMHLW; therefore, medical costs may change in the future; (6) this study examines cross-sectional data from one incorporated medical institution in a rural area of Japan and the cost benefits of Zaitaku care may be increased when compared with lower categorized care clinics and hospitals. These results can inform evidence based policy making for private and/or public medical insurance systems. Cost is important but other factors such as patient and family satisfaction and quality of care also should be investigated further.

## Conclusions

Although the Zaitaku physician-led home visit team care costs more than hospital care for short-term care (less than 10 days), costs may be lower than hospital care for long-term care (more than 30 days) at the end of life. Medical costs at a Zaitaku care at long-term was 82.1% lower than that care at short-term ($155.8 vs. $868.0, *p* < 0.05). Medical costs of Zaitaku care in the long-term (more than 30 days) were 16.7% lower than that of hospital care ($155.8 vs. $187.4, *p* = 0.055). To fulfil patients’ wishes to spend the end of life at home and promote a community-based integrated care system, physician-led home visit team care is a promising option for medical care with possible cost benefits when implemented earlier in the end-of-life period. This report is the first study evaluating the medical costs of the physician-led home visits team care at the end of life care.

## Additional files

Additional file 1: Patient profiles with Zaitaku care less than 30 days before dying. (PPTX 113 kb)
Additional file 2: Patient profiles with Zaitaku care more than 30 days before dying. (PPTX 110 kb)
Additional file 3: Average medical costs per day for hospital care in Japan (US$/day). (PPTX 69 kb)

## Abbreviations

JMHLW: Japanese Ministry of Health, Labor and Welfare
LTCI: Long-Term Care Insurance
NHI: National Health Insurance
U.K.: United Kingdom
U.S.: United States
