# Supplementary material for: Cost of physician-led home visit care (Zaitaku care) compared with hospital care at the end of life in Japan
Source: BMC Health Serv Res. 2017 Jan 17;17:40. doi: 10.1186/s12913-016-1961-x (PMC5240473; doi:10.1186/s12913-016-1961-x)
Supplement: Additional file 1: — Patient profiles with Zaitaku care less than 30 days before dying. (PPTX 113 kb) [file 12913_2016_1961_MOESM1_ESM.pptx]

## Slide 1
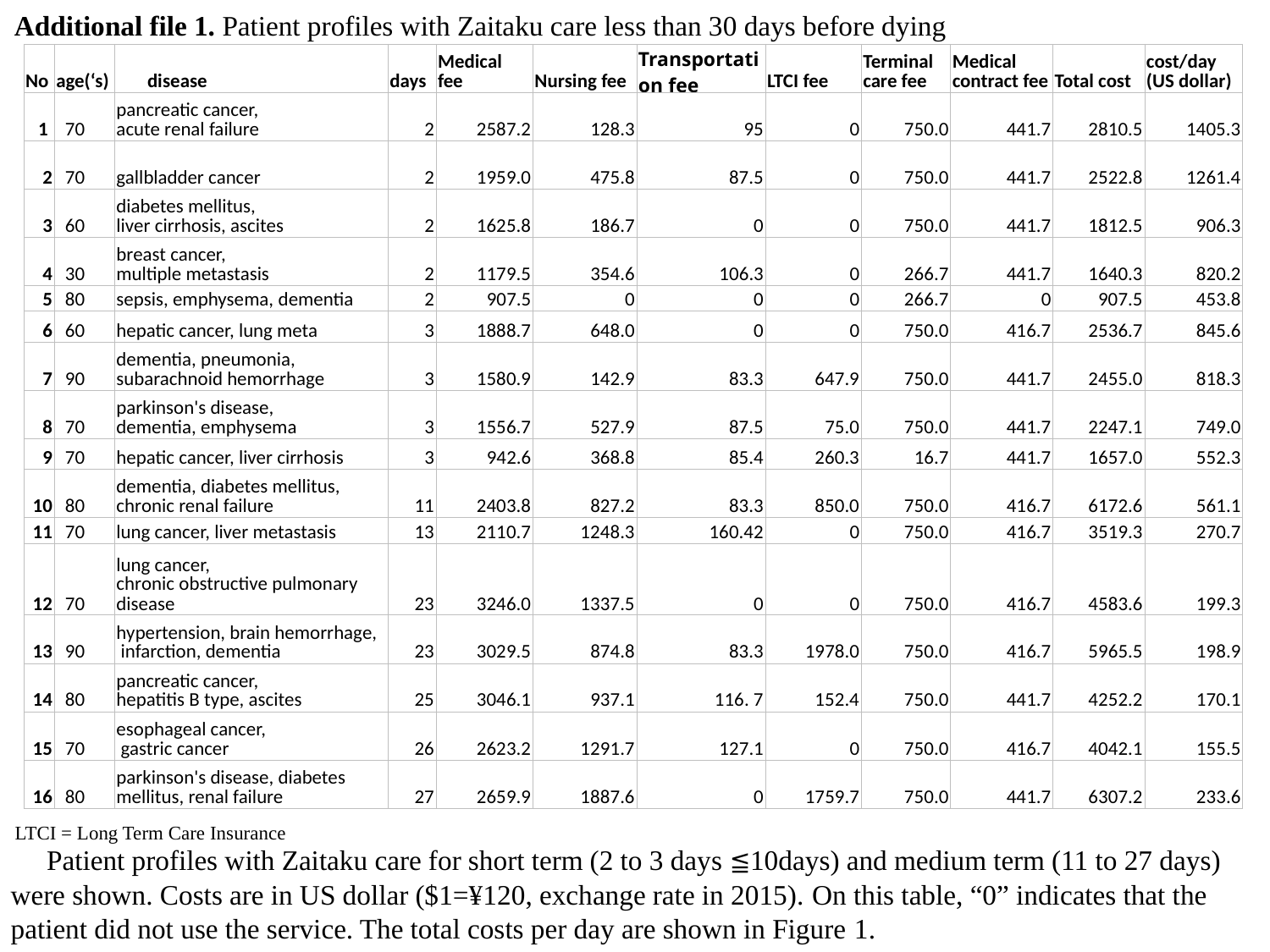

Additional file 1. Patient profiles with Zaitaku care less than 30 days before dying
| No | age(‘s) | disease | days | Medical fee | Nursing fee | Transportation fee | LTCI fee | Terminal care fee | Medical contract fee | Total cost | cost/day (US dollar) |
| --- | --- | --- | --- | --- | --- | --- | --- | --- | --- | --- | --- |
| 1 | 70 | pancreatic cancer, acute renal failure | 2 | 2587.2 | 128.3 | 95 | 0 | 750.0 | 441.7 | 2810.5 | 1405.3 |
| 2 | 70 | gallbladder cancer | 2 | 1959.0 | 475.8 | 87.5 | 0 | 750.0 | 441.7 | 2522.8 | 1261.4 |
| 3 | 60 | diabetes mellitus, liver cirrhosis, ascites | 2 | 1625.8 | 186.7 | 0 | 0 | 750.0 | 441.7 | 1812.5 | 906.3 |
| 4 | 30 | breast cancer, multiple metastasis | 2 | 1179.5 | 354.6 | 106.3 | 0 | 266.7 | 441.7 | 1640.3 | 820.2 |
| 5 | 80 | sepsis, emphysema, dementia | 2 | 907.5 | 0 | 0 | 0 | 266.7 | 0 | 907.5 | 453.8 |
| 6 | 60 | hepatic cancer, lung meta | 3 | 1888.7 | 648.0 | 0 | 0 | 750.0 | 416.7 | 2536.7 | 845.6 |
| 7 | 90 | dementia, pneumonia, subarachnoid hemorrhage | 3 | 1580.9 | 142.9 | 83.3 | 647.9 | 750.0 | 441.7 | 2455.0 | 818.3 |
| 8 | 70 | parkinson's disease, dementia, emphysema | 3 | 1556.7 | 527.9 | 87.5 | 75.0 | 750.0 | 441.7 | 2247.1 | 749.0 |
| 9 | 70 | hepatic cancer, liver cirrhosis | 3 | 942.6 | 368.8 | 85.4 | 260.3 | 16.7 | 441.7 | 1657.0 | 552.3 |
| 10 | 80 | dementia, diabetes mellitus, chronic renal failure | 11 | 2403.8 | 827.2 | 83.3 | 850.0 | 750.0 | 416.7 | 6172.6 | 561.1 |
| 11 | 70 | lung cancer, liver metastasis | 13 | 2110.7 | 1248.3 | 160.42 | 0 | 750.0 | 416.7 | 3519.3 | 270.7 |
| 12 | 70 | lung cancer, chronic obstructive pulmonary disease | 23 | 3246.0 | 1337.5 | 0 | 0 | 750.0 | 416.7 | 4583.6 | 199.3 |
| 13 | 90 | hypertension, brain hemorrhage, infarction, dementia | 23 | 3029.5 | 874.8 | 83.3 | 1978.0 | 750.0 | 416.7 | 5965.5 | 198.9 |
| 14 | 80 | pancreatic cancer, hepatitis B type, ascites | 25 | 3046.1 | 937.1 | 116. 7 | 152.4 | 750.0 | 441.7 | 4252.2 | 170.1 |
| 15 | 70 | esophageal cancer, gastric cancer | 26 | 2623.2 | 1291.7 | 127.1 | 0 | 750.0 | 416.7 | 4042.1 | 155.5 |
| 16 | 80 | parkinson's disease, diabetes mellitus, renal failure | 27 | 2659.9 | 1887.6 | 0 | 1759.7 | 750.0 | 441.7 | 6307.2 | 233.6 |
LTCI = Long Term Care Insurance
 Patient profiles with Zaitaku care for short term (2 to 3 days ≦10days) and medium term (11 to 27 days) were shown. Costs are in US dollar ($1=¥120, exchange rate in 2015). On this table, “0” indicates that the patient did not use the service. The total costs per day are shown in Figure 1.
