# Supplementary material for: Cost of physician-led home visit care (Zaitaku care) compared with hospital care at the end of life in Japan
Source: BMC Health Serv Res. 2017 Jan 17;17:40. doi: 10.1186/s12913-016-1961-x (PMC5240473; doi:10.1186/s12913-016-1961-x)
Supplement: Additional file 2: — Patient profiles with Zaitaku care more than 30 days before dying. (PPTX 110 kb) [file 12913_2016_1961_MOESM2_ESM.pptx]

## Slide 1
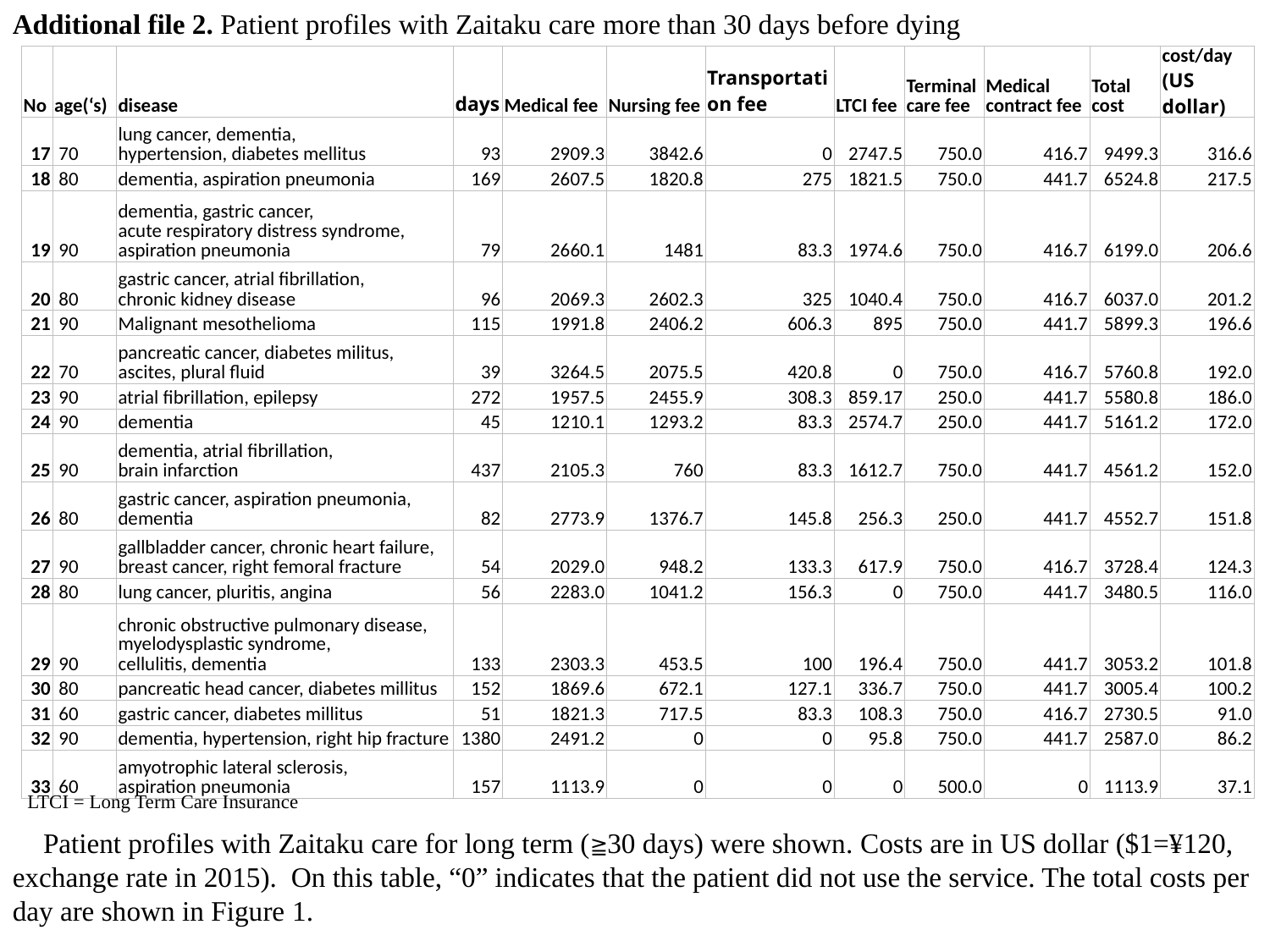

Additional file 2. Patient profiles with Zaitaku care more than 30 days before dying
| No | age(‘s) | disease | days | Medical fee | Nursing fee | Transportation fee | LTCI fee | Terminal care fee | Medical contract fee | Total cost | cost/day (US dollar) |
| --- | --- | --- | --- | --- | --- | --- | --- | --- | --- | --- | --- |
| 17 | 70 | lung cancer, dementia, hypertension, diabetes mellitus | 93 | 2909.3 | 3842.6 | 0 | 2747.5 | 750.0 | 416.7 | 9499.3 | 316.6 |
| 18 | 80 | dementia, aspiration pneumonia | 169 | 2607.5 | 1820.8 | 275 | 1821.5 | 750.0 | 441.7 | 6524.8 | 217.5 |
| 19 | 90 | dementia, gastric cancer, acute respiratory distress syndrome, aspiration pneumonia | 79 | 2660.1 | 1481 | 83.3 | 1974.6 | 750.0 | 416.7 | 6199.0 | 206.6 |
| 20 | 80 | gastric cancer, atrial fibrillation, chronic kidney disease | 96 | 2069.3 | 2602.3 | 325 | 1040.4 | 750.0 | 416.7 | 6037.0 | 201.2 |
| 21 | 90 | Malignant mesothelioma | 115 | 1991.8 | 2406.2 | 606.3 | 895 | 750.0 | 441.7 | 5899.3 | 196.6 |
| 22 | 70 | pancreatic cancer, diabetes militus, ascites, plural fluid | 39 | 3264.5 | 2075.5 | 420.8 | 0 | 750.0 | 416.7 | 5760.8 | 192.0 |
| 23 | 90 | atrial fibrillation, epilepsy | 272 | 1957.5 | 2455.9 | 308.3 | 859.17 | 250.0 | 441.7 | 5580.8 | 186.0 |
| 24 | 90 | dementia | 45 | 1210.1 | 1293.2 | 83.3 | 2574.7 | 250.0 | 441.7 | 5161.2 | 172.0 |
| 25 | 90 | dementia, atrial fibrillation, brain infarction | 437 | 2105.3 | 760 | 83.3 | 1612.7 | 750.0 | 441.7 | 4561.2 | 152.0 |
| 26 | 80 | gastric cancer, aspiration pneumonia, dementia | 82 | 2773.9 | 1376.7 | 145.8 | 256.3 | 250.0 | 441.7 | 4552.7 | 151.8 |
| 27 | 90 | gallbladder cancer, chronic heart failure, breast cancer, right femoral fracture | 54 | 2029.0 | 948.2 | 133.3 | 617.9 | 750.0 | 416.7 | 3728.4 | 124.3 |
| 28 | 80 | lung cancer, pluritis, angina | 56 | 2283.0 | 1041.2 | 156.3 | 0 | 750.0 | 441.7 | 3480.5 | 116.0 |
| 29 | 90 | chronic obstructive pulmonary disease, myelodysplastic syndrome, cellulitis, dementia | 133 | 2303.3 | 453.5 | 100 | 196.4 | 750.0 | 441.7 | 3053.2 | 101.8 |
| 30 | 80 | pancreatic head cancer, diabetes millitus | 152 | 1869.6 | 672.1 | 127.1 | 336.7 | 750.0 | 441.7 | 3005.4 | 100.2 |
| 31 | 60 | gastric cancer, diabetes millitus | 51 | 1821.3 | 717.5 | 83.3 | 108.3 | 750.0 | 416.7 | 2730.5 | 91.0 |
| 32 | 90 | dementia, hypertension, right hip fracture | 1380 | 2491.2 | 0 | 0 | 95.8 | 750.0 | 441.7 | 2587.0 | 86.2 |
| 33 | 60 | amyotrophic lateral sclerosis, aspiration pneumonia | 157 | 1113.9 | 0 | 0 | 0 | 500.0 | 0 | 1113.9 | 37.1 |
LTCI = Long Term Care Insurance
 Patient profiles with Zaitaku care for long term (≧30 days) were shown. Costs are in US dollar ($1=¥120, exchange rate in 2015). On this table, “0” indicates that the patient did not use the service. The total costs per day are shown in Figure 1.
