# Supplementary material for: Cost of physician-led home visit care (Zaitaku care) compared with hospital care at the end of life in Japan
Source: BMC Health Serv Res. 2017 Jan 17;17:40. doi: 10.1186/s12913-016-1961-x (PMC5240473; doi:10.1186/s12913-016-1961-x)
Supplement: Additional file 3: — Average medical costs per day for hospital care in Japan (US$/day). (PPTX 69 kb) [file 12913_2016_1961_MOESM3_ESM.pptx]

## Slide 1
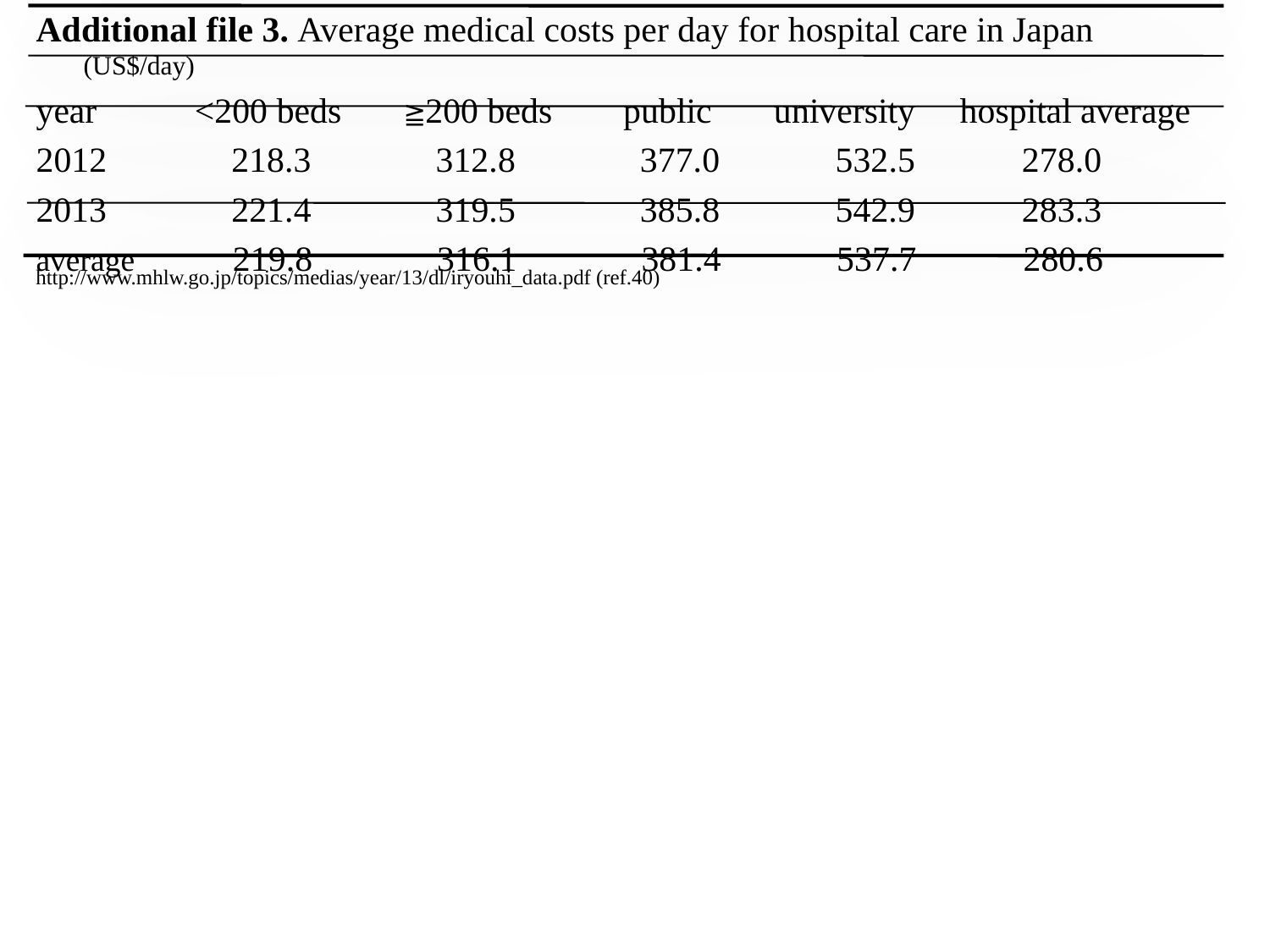

Additional file 3. Average medical costs per day for hospital care in Japan (US$/day)
year <200 beds ≧200 beds public university hospital average
2012 218.3 312.8 377.0 532.5 278.0
 221.4 319.5 385.8 542.9 283.3
average 219.8 316.1 381.4 537.7 280.6
http://www.mhlw.go.jp/topics/medias/year/13/dl/iryouhi_data.pdf (ref.40)
